# Association Duty of the Hour

**Published:** 1895-12

**Authors:** 


### ASSOCIATION DUTY OF THE HOUR.

EVERY veterinary association in the land has its influence and power for good, and failure to exercise this strength has lost for our cause many battles for position and place in every community where these organizations exist. Every centre of intelligence and progress, where three or more veterinarians are gathered together, without a community of purpose, without frequent conferences one with another, without an organization where every public question of sanitary importance is considered, are losing golden opportunities for themselves and strewing the pathway of their successors with difficulties and obstructions that will be hard to overcome. The little plant of indifference grows while we sleep and wait; its roots find rich soil while we forget our duty to our calling, and when well rooted, with all its rootlets following as many diverse ramifications, attains a sturdy growth that baffles our best efforts for its destruction. The hour of our greatest trial in veterinary spheres has passed, the storm has spent its greatest force, and while we have all felt sorely the trials and tribulations of our lot, we have not been alone; nor have the grave lessons which this season of depression brought been without their value, and the silver lining of the blackened and obscured horizon points soon to a noonday sun of prosperity. It has opened for us the great sanitary field of veterinary police; it has brought us face to face with a better conception of our limitless field of labor, and trolleys, bicycles, and motor forces for our carriages and drays are only sudden changes that will broaden the territory of our usefulness.

The demoralized horse-market that fell at the highest boom from the inflation that follows every misguided occupation, that led to the most reckless breeding of horses ever witnessed in any intelligent nation, and that produced a nearly limitless number of horses good for nothing else but street-car service, and at the best an extravagant form of motor power for even this service, will teach a better lesson than all the preaching for wiser breading and greater care. The production of a better horse, for every purpose required, will follow as surely as night follows day, and values will increase to a more permanent, high standard than was ever destined to follow such a dangerous system that flourished and prevailed for so long a time.

Every hamlet, village, town, and city is learning the daily lesson taught that the sources of their food and drink cannot be polluted at their tributaries and still be without danger at the place of delivery for consumption. Every intelligent, progressive community is well considering the lesson that preventive medicine is the acme of our science; and how much shall we as members of a learned calling, how much shall we as representative sanitarians and citizens, help to impart this lesson well? How much shall we in our organizations help our people to solve these all-important demands of the hour will be measured by the success we attain in creating for our people a just and fair conception of our usefulness and by the extent we contribute to the relief of our people from these dangers. Let our associations be up and doing; let them multiply wherever they can; let them meet together often for deliberation and considerate discussion of these problems; let this present season be a memorable one in the annals of our progress and every movement for higher, truer, sanitary knowledge feel the force of our sustenance and aid, and the sunshine of prosperity will again lighten our sphere of work, broaden our conceptions of the great responsibilities we owe to our people.

There will be fewer of our number stray from the fold of honesty and integrity to strew the pathway of deception and dishonor with wrecked characters in their efforts to win from our people the fruits of their toil by efforts at deceiving and robbing the innocent and unwary through the sale of nostrums, cure-alls, and remedies of no value; wholly depending upon lying, false testimonials, and the artful practices always pursued by the humbugs and dishonest of every community, which always end disastrously to every profession and calling in the shaken confidence and unbelief that forever rankle in the minds of those who are robbed and deceived under the thin disguise of some great discovery.

